# Different cell compositions and a novel somatic *KCNJ5* variant found in a patient with bilateral adrenocortical adenomas secreting aldosterone and cortisol

**DOI:** 10.3389/fendo.2023.1068335

**Published:** 2023-03-07

**Authors:** Liling Zhao, Jinjing Wan, Yujun Wang, Wenjun Yang, Qi Liang, Jinrong Wang, Ping Jin

**Affiliations:** ^1^ Department of Endocrinology, The Third Xiangya Hospital, Central South University, Changsha, Hunan, China; ^2^ Department of Radiology, The Third Xiangya Hospital, Central South University, Changsha, Hunan, China; ^3^ Department of Urology, The Third Xiangya Hospital, Central South University, Changsha, Hunan, China

**Keywords:** primary aldosteronism, Cushing’s syndrome, KCNJ5 gene, adrenal vein sampling, CYP11B1, CYP11B2, aldosterone- and cortisol-producing adenoma

## Abstract

**Introduction:**

This study aimed to explore the possible pathogenesis of a rare case of co-existing Cushing’s syndrome (CS) and primary aldosteronism (PA) caused by bilateral adrenocortical adenomas secreting aldosterone and cortisol, respectively.

**Methods:**

A 41-year-old Chinese woman with severe hypertension and hypokalemia for 5 and 2 years, respectively, was referred to our hospital. She had a Cushingoid appearance. Preoperative endocrinological examinations revealed autonomous cortisol and aldosterone secretion. Computed tomography revealed bilateral adrenal adenomas. Subsequently, adrenal vein sampling and sequential left and right partial adrenalectomy indicated the presence of a left aldosterone-producing tumor and a right cortisol-producing tumor. Pathological examination included immunohistochemical analysis of the resected specimens. Secretions of aldosterone and cortisol were observed both *in vivo* and *in vitro*. Further, whole-exome sequencing was performed for DNA that was extracted from peripheral blood leukocytes and bilateral adrenal adenomas in order to determine whether the patient had relevant variants associated with PA and CS.

**Results:**

Immunohistochemical staining revealed that the left adenoma primarily comprised clear cells expressing CYP11B2, whereas the right adenoma comprised both eosinophilic compact and clear cells expressing CYP11B1. The mRNA levels of steroidogenic enzymes (including CYP11B1 and CYP17A1) were high in the right adenoma, whereas CYP11B2 was highly expressed in the left adenoma. A novel somatic heterozygous missense variant—*KCNJ5* c.503T > G (p.L168R)—was detected in the left adrenal adenoma, but no other causative variants associated with PA and CS were detected in the peripheral blood or right adrenocortical adenoma. In the primary cell culture of the resected hyperplastic adrenal adenomas, verapamil and nifedipine, which are two calcium channel blockers, markedly inhibited the secretion of both aldosterone and cortisol.

**Conclusion:**

We present an extremely rare case of bilateral adrenocortical adenomas with distinct secretion of aldosterone and cortisol. The heterogeneity of the tumor cell compositions of aldosterone- and cortisol-producing adenoma (A/CPA) and somatic mutation of *KCNJ5* may have led to different hormone secretions in the bilateral adrenal adenomas.

## Introduction

Primary aldosteronism (PA) is considered the most common cause of secondary hypertension, and it has been reported to be present in at least 5%–10% of patients with hypertension (1). PA is caused by excessive production of aldosterone and inhibition of renin activity, resulting in hypertension and hypokalemic alkalosis. Moreover, PA is commonly caused by aldosterone-producing adenomas (APAs), idiopathic adrenal hyperplasia, or rare glucocorticoid-remediable aldosteronism. In 1977, Hogan et al. reported the first case of PA with significant cortisol auto-secretion, which was considered to be caused by an aldosterone- and cortisol-producing adenoma (A/CPA) (2). Currently, A/CPA is recognized as a subtype of PA because it is frequently detected when screening for PA. Recent studies have reported that the prevalence of subclinical Cushing’s syndrome (CS) may be high (21%–26.8%) in patients with APA (3, 4), and 5%–21% of adrenal tumors are A/CPA (3, 5, 6). As reported previously, PA associated with cortisol autonomous secretion can be classified into the following types: 1) a single ipsilateral adrenal adenoma secreting both cortisol and aldosterone simultaneously; 2) two adenomas on ipsilateral adrenal glands secreting aldosterone and cortisol separately; and 3) two adenomas, one on each side of the adrenal gland, secreting aldosterone and cortisol separately (3). Most patients had adenomas secreting both cortisol and aldosterone simultaneously, and the cases of bilateral adenomas secreting different hormones independently are extremely rare.

Over the past few years, somatic mutations have been reported to be associated with the development of A/CPA. To date, more than eight genes have been reported to be associated with APAs, including *KCNJ5*, *CACNA1D*, *ATP1A1*, *ATP2B3*, *CACNA1H*, *CLCN2*, *CTNNB1*, and/or *GNAQ/11* (7, 8), representing >50% of sporadic APAs. Of these, mutations in *KCNJ5* are the most common, with the *KCNJ5* mutation rate in APA reportedly being approximately 40% in Western countries (9) and 60%–70% in Asian countries (10, 11). A recent study reported that among Chinese patients with APAs, the mutation rates of *KCNJ5*, *ATP1A1*, *ATP2B3*, and *CACNA1D* are 77%, 2%, 0.5%, and 0.5%, respectively Page: 4 (11). 

Similarly, somatic gene mutations have been identified in approximately 50% of the cortisol-producing adenomas (CPAs). The affected genes include *PRKACA*, *GNAS*, *PRKAR1A*, and *CTNNB1*, with *PRKACA* being the most frequently mutated gene (12, 13). Furthermore, somatic *KCNJ5* and *PRKACA* mutations have been found in patients with A/CPA (14, 15).

In this study, we aimed to explore the possible pathogenesis of a patient with bilateral adrenal adenomas secreting aldosterone and cortisol, respectively.

## Materials and methods

### Ethics statement

This study was approved by the Institutional Ethics Committee of the Third Xiangya Hospital. After obtaining written informed consent, we collected peripheral blood samples as well as resected adrenocortical adenomas from the patient.

### Adrenal vein sampling (AVS)

Bilaterally simultaneous AVS techniques with cosyntropin (synthetic adrenocorticotropic hormone 1–24) stimulation were performed as described in a previous study (16). The detailed steps were as follows: after successful placement of bilateral adrenal veins, blood samples were collected at baseline and at 10 minutes and 5 minutes before cosyntropin stimulation as well as at 10 minutes and 20 minutes after the stimulation. Overall, 125 μg of cosyntropin was intravenously injected; subsequently, 125 μg of cosyntropin was infused continuously for 1 hour. Further, cortisol and aldosterone levels were measured using the blood samples.

### Whole-exome sequencing

To determine whether the patient had relevant gene variants associated with PA and CS, we performed whole-exome sequencing of DNA extracted from peripheral blood leukocytes and resected bilateral adrenocortical adenomas. The isolated DNA was sheared on a Bioruptor UCD-200 (Diagenode) with a size distribution peak of approximately 200 bp. The samples were diluted, loaded, and sequenced on the HiSeq2500 platform (Illumina, San Diego, CA). Further, exome data processing and variant annotation were performed as described in a previous study (17). The variants were interpreted according to the standards of the American College of Medical Genetics (ACMG) and categorized as follows: pathogenic, likely pathogenic, variants of uncertain significance, likely benign, or benign.

### *In silico* analysis

The effects of single nucleotide variants were predicted using SIFT (http://sift.jcvi.org), PolyPhen-2 (http://genetics.bwh.harvard.edu/pph2), MutationTaster (http://www.mutationtaster.org), and PROVEAN (http://provean.jcvi.org/index.php) programs. Further, KCNJ5 amino acids across different species were aligned using AlignX software (Invitrogen).

### Immunohistochemical analysis

Immunohistochemical analysis was performed using an En Vision detection kit (Dako). The antibodies and dilutions were as follows: Ki-67 (1:500, OriGene), CYP11B1 (1:200, EMD Millipore), and CYP11B2 (1:500, EMD Millipore). Normal adrenal medulla was used as negative tissue control.

### Primary cell culture of the adrenal tumors

The resected bilateral adrenal adenoma tissues (wet weight, 1 g) were washed twice with Dulbecco’s modified eagle medium (DMEM), cut into small pieces, and dispersed by incubation in DMEM containing 2% collagenase I for 20 minutes at 37°C. Primary cell culture of the adrenal adenomas was performed as described previously (18). On day 4, when the cells grew to 80% confluence, they were treated with verapamil (10 μM, Sigma-Aldrich) and nifedipine (10 μM, Sigma-Aldrich) or with the vehicle for 24 hours. The culture medium was collected for measuring aldosterone and cortisol, and the concentrations of aldosterone (Sinbe Diagnostic, China) and cortisol (Roche Diagnostics GmbH, Germany) in the medium were directly measured *via* chemiluminescence.

### RNA isolation and real-time quantitative PCR

To clarify the expression of steroidogenic enzymes for the synthesis of aldosterone and cortisol in bilateral adrenal adenomas, we examined the mRNA levels of steroidogenic enzymes, including steroidogenic acute regulatory protein (STAR), 17α-hydroxylase (CYP17A1), 11β-hydroxylase (CYP11B1), and aldosterone synthase (CYP11B2), in the bilateral adrenal adenomas and other adrenal adenomas, including

five APAs, five CPAs, and three nonfunctional adenomas. Further, the cells and tissues were lysed using TRIzol Reagent (Sigma-Aldrich), and total RNA was isolated in accordance with the standard protocol provided by the manufacturer. The cDNA was synthesized from 1 μg of total RNA using a high-capacity cDNA reverse transcription kit (Applied Biosystems). PCR amplification for the expression of STAR, CYP17A1, CYP11B1, and CYP11B2 was conducted using the LightCycler 480 SYBR Green I Master (Roche Applied Science) on a LightCycler 480 PCR system. Moreover, relative gene expression levels were determined with the cycle threshold value and were normalized against the expression of the human glyceraldehyde-3-phosphate de-hydrogenase (GAPDH) gene. The primers were used in accordance with a previous study (18).

### Statistical analysis

The data were presented as mean ± standard deviation for primary cell culture and qPCR tests. Two groups were compared using the Student *t*-test, and multiple groups were analyzed by one-way analysis of variance (GraphPad Prism, La Jolla, CA, USA). *P* < 0.05 was considered statistically significant.

## Results

### Clinical characteristics

**Figure 1:**
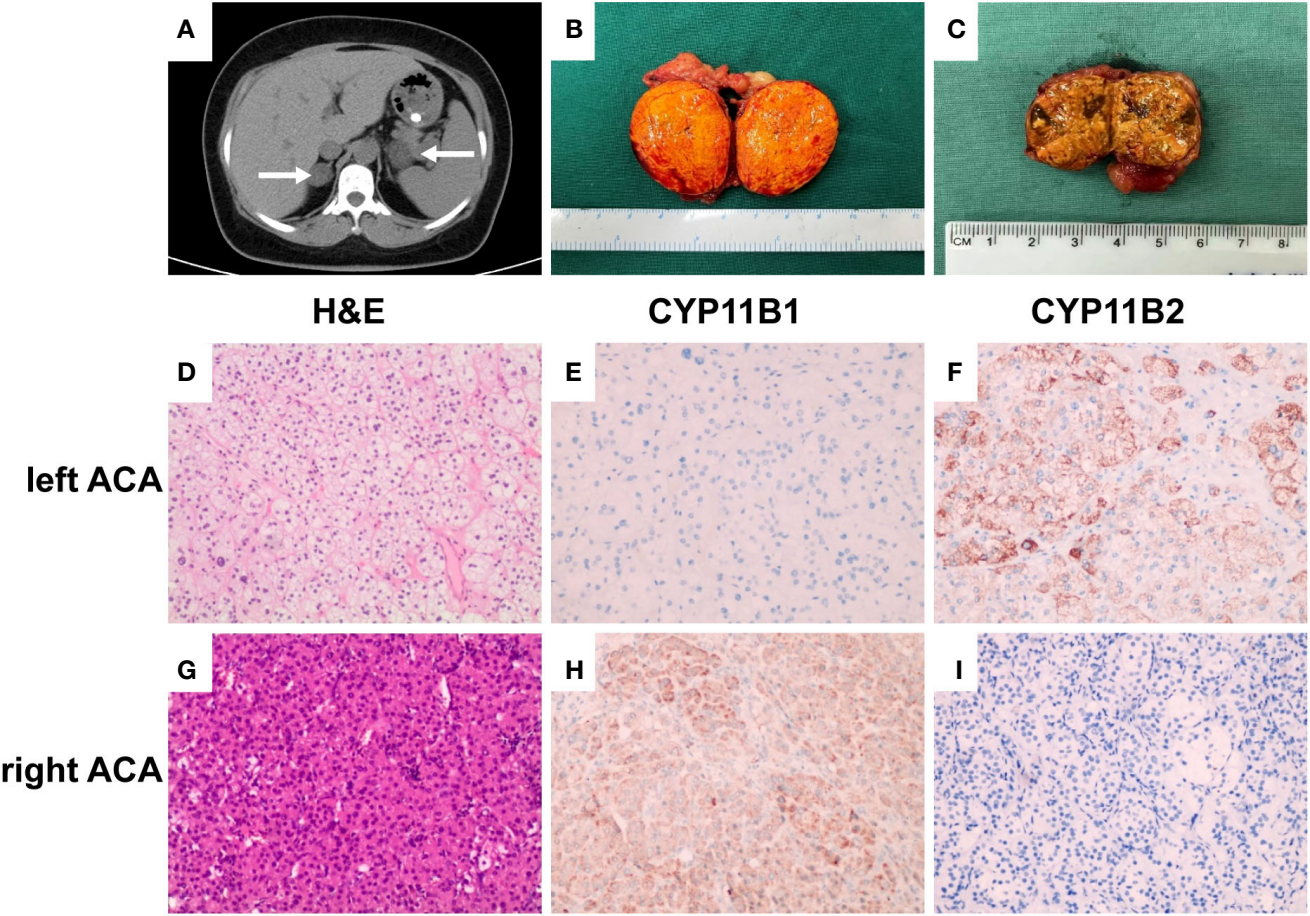
Enhanced computerized tomography (CT), pathological and immunohistochemical findings. **(A)** CT scan revealed bilateral adrenocortical adenomas, with the left adenoma of 38 × 28 mm and the right adenoma of 25 × 21 mm (white arrows). **(B)** Gross pathology of the resected left adrenal tumor showing a bright yellow well-circumscribed mass arising from the cortex. **(C)** Gross pathology of the resected right adrenal tumor showing a brownish yellow, relatively solid area. **(D, G)** H&E stain results, (×200); **(E, F, H, I)** immunostaining (×200). Left adrenocortical adenoma (left ACA, predominately secreted aldosterone) consisting primarily of clear cells, CYP11B1 (−) and CYP11B2 (+). Right adrenocortical adenoma (right ACA, predominately secreting cortisol) was composed of both eosinophilic compact cells and clear cells, with the former accounting for 60%, CYP11B1 (+) and CYP11B2 (−).

A 41-year-old Chinese woman with severe hypertension and hypokalemia for 5 and 2 years, respectively, was referred to our hospital. She was diagnosed with hypertension 5 years ago and was administered telmisartan (40 mg qd) and amlodipine besylate (5 mg qd), but her blood pressure remained poorly controlled. Two years ago, she was diagnosed with hypokalemia (2.1 mmol/L) because of fatigue and was administered potassium intermittently. She was also diagnosed with new-onset diabetes during that time, but she was not taking any medication. Ten days ago, she was referred to our hospital because of severe shortness of breath after experiencing cold. Based on her physical examination, her blood pressure (BP) was 187/130 mmHg, height was 148 cm, weight was 64 kg, and body mass index was 29.2 kg/cm^2^. Notably, she had a Cushingoid appearance with a moon face, a buffalo hump, and centripetal obesity; however, no purplish abdominal striae were found. Laboratory examination at admission revealed marked hypokalemia with a high urinary potassium level (80.25 mmol/24 h). Moreover, she had elevated levels of aldosterone, suppressed renin activity, and a significantly high aldosterone-to-renin ratio (ARR) of 1623.3 (**Table 1**). Furthermore, the captopril challenge test failed to suppress aldosterone secretion. Additionally, she presented with elevated levels of cortisol and 24-hour urinary free cortisol (UFC), loss of normal diurnal rhythm, and suppressed levels of adrenocorticotropic hormone (ACTH) (**Table 1**). Moreover, serum cortisol remained unsuppressed after overnight 1-mg dexamethasone suppression test (DST) and high-dose (8 mg) dexamethasone suppression test (**Table 1**). Based on these results, the patient was diagnosed with co-existing ACTH-independent CS and primary aldosteronism. Moreover, serum catecholamine, metanephrine, methoxynorepinephrine, follicle-stimulating hormone, luteinizing hormone, estradiol, testosterone, and dehydroepiandrosterone sulfate levels were normal. Furthermore, glycated hemoglobin (HbA1c) level was 7.7%, creatinine level was 113 μmol/L (RR, 41–85 μmol/L), urinary microalbumin-to-creatinine ratio was 590.7 mg/g (RR, 0–30 mg/g), and brain natriuretic peptide level was 6850.8 pg/ml (RR, 0–500 pg/ml). ECG findings indicated sinus tachycardia with left ventricular high voltage. Chest X-ray revealed an enlarged heart, and the cardiothoracic ratio was 0.74. Fundus examination demonstrated 1:2 arteriovenous pressure traces and a small amount of fundus exudates, indicating changes in the fundus of patients with hypertension. Further, the results of echocardiography indicated a large left atrium and thickened left ventricular wall; moreover, the percentages of ejection fraction and fractional shortening were 50% and 32%, respectively. Computerized tomography scan revealed bilateral adrenocortical adenomas, with the left adenoma of 38 × 28 mm and the right adenoma of 25 × 21 mm (**Figure 1A**).

**Table 1:** Clinical data and treatments before and after operations.

Index	Before operation	After operation of the left adenoma (One month)	After operation of the right adenoma (Three months)	RR
Plasma potassium (mmol/l)	2.8	4.0	4.7	3.5–5.5
Aldosterone (pg/ml)	974.1	108.5	95.6	65.0–150.0
Plasma renin activity (ng/ml/h)	0.06	6.2	3.1	0.9–6.5
ARR	1623.3	1.7	3.1	<30
Cortisol basal (μg/dl)	22.9	19.8	8.9	4.8–19.5
Cortisol after 1-mg DST (μg/dl)	22.6	16.5	0.7	<1.8
Cortisol after HDDST (μg/dl)	20.4	NA	NA	
ACTH (pg/ml)	<1	3.9	36.9	7.2–63.3
UFC basal (μg/day)	186.9	114.6	20.1	3.5–45.0
UFC after 1-mg DST (μg/day)	145.6	108.7	NA	3.5-45.0
UFC after HDDST (μg/day)	120.3	NA	NA	3.5–45.0
Hypoglycemic therapy	OHA + INS	OHA	NO	
Antihypertensive agents	YES (Four drugs)	Yes (One drug)	NO	

ARR, aldosterone-to-renin ratio; 1-mg DST, overnight 1-mg dexamethasone suppression test; ACTH, adrenocorticotropic hormone; UFC, urinary free cortisol; HDDST, high-dose dexamethasone suppression test; OHA, oral hypoglycemic agents; NA, not available; RR: reference range.

Bilateral AVS revealed that the aldosterone concentration in the left adrenal vein (LAV) was remarkably higher than that in the right adrenal vein (RAV) and the inferior vena cava (IVC), indicating aldosterone overproduction from the left side. Moreover, the cortisol concentration in RAV was 3.1–9.7 times higher than that in the LAV, indicating cortisol overproduction in the left side (**Table 2**). Furthermore, significant adrenal-to-IVC gradient of cortisol enabled successful catheterization on both sides.

**Table 2:** Results of AVS.

	Aldosterone (pg/mL)						Cortisol (μg/dL)					
	LAV	RAV	IVC	L/R	L/I	R/I	LAV	RAV	IVC	R/L	L/I	R/I
−10’	93590.0	1600.7	1094.6	58.5	85.5	1.5	285.0	1032.4	24.8	3.6	11.5	41.6
−5’	94576.0	1426.3	1149.6	66.3	82.3	1.2	295.0	932.8	36.6	3.2	8.1	25.5
10’ post ACTH	132587.0	5716.0	1202.9	23.2	110.2	4.8	427.0	4141.7	36.9	9.7	11.6	112.2
20’ post ACTH	152230.0	5863.0	1336.4	26.0	113.9	4.4	1390.0	4297.2	30.6	3.1	45.4	140.4

AVS, adrenal vein sampling; LAV, left adrenal vein; RAV, right adrenal vein; IVC inferior vena cava; L/R, LAV: RAV ratio; L/I, LAV: IVC ratio; R/I, RAV: IVC ratio; R/L, RAV: LAV ratio; ACTH, adrenocorticotropic hormone.

### Treatment and follow-up

The patient was treated with antihypertensive agents (spironolactone 100 mg qd, nifedipine 60 mg qd, metoprolol 47.5 mg qd, prazosin 2 mg q8h, and sacubitril valsartan sodium 50 mg bid) and hypoglycemic therapy (insulin degludec and insulin aspart injection 16 IU qd, linagliptin 5 mg qd, and acarbose 50 mg tid). As the left adenoma was larger than the right adenoma and there was a possibility of hypersecretion of aldosterone from the left tumor, the patient underwent left laparoscopic adrenalectomy. One month after the left partial adrenalectomy, her BP decreased significantly, and she only needed to take 5 mg amlodipine daily to maintain BP at 130–140/90 mmHg. Meanwhile, she stopped receiving insulin therapy and took only one oral hypoglycemic agent. Further, her aldosterone level significantly decreased, renin activity increased, and ARR and serum potassium levels normalized (**Table 1**). However, serum cortisol and 24-hour UFC still exceeded the normal range. Further, serum cortisol remained unsuppressed after 1-mg DST, indicating that cortisol over-secretion from the right adrenal adenoma persisted. Subsequently, she underwent right laparoscopic adrenalectomy. One month after the second surgery, her BP, blood glucose level, and serum potassium level were found to be normalized without any medication. Replacement therapy with prednisone (5 mg daily) was provided to avoid adrenal insufficiency. Three months later, the patient stopped taking prednisone, her BP and blood glucose levels remained normal, cortisol and ACTH levels returned to the normal ranges, and her condition could be suppressed with a low-dose DST (**Table 1**).

### Immunohistochemical analysis

Pathological examination revealed that the left adrenal mass was a golden-yellow adenoma (**Figure 1B**) mainly composed of clear cells on hematoxylin/eosin stained sections (**Figure 1D**). Immunohistochemical analysis revealed cytoplasm immunoreactivity for aldosterone synthase (CYP11B2) with no expression of 11beta-hydroxylase 1 (CYP11B1; **Figures 1E, F**). Pathological examination revealed that the right adrenal tumor was brownish yellow and relatively solid (**Figure 1C**). This mass was composed of both eosinophilic compact and clear cells, with the former accounting for 60% (**Figure 1G**). Immunohistochemical analysis also revealed cytoplasm immunoreactivity for CYP11B1 with no expression of CYP11B2 (**Figures 1H, I**). These results confirmed that the patient had a co-existing left aldosterone-producing adenoma and right cortisol-producing adrenal adenoma, which was consistent with the results of AVS.

### mRNA expression of STAR, CYP17A1, CYP11B1, and CYP11B2

**Figure 2:**
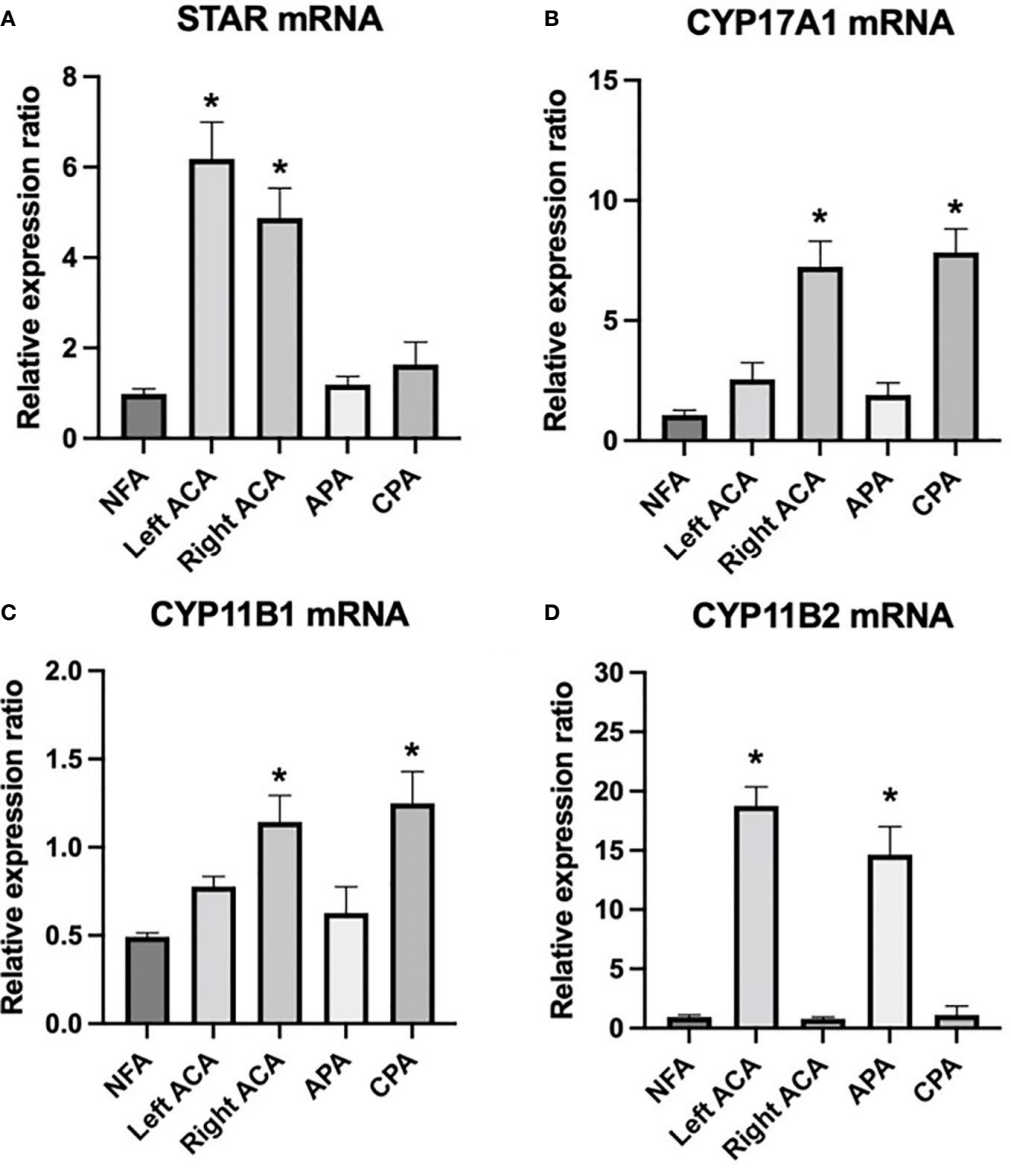
mRNA expression of steroidogenic enzymes *STAR*, *CYP17A1*, *CYP11B1*, and *CYP11B2* in the bilateral adrenal adenomas of the patient and other adrenal adenomas. **(A)** *STAR* was highly expressed in both bilateral adenomas, **(B, C)** *CYP11B1 and CYP17A1* were highly expressed in right adenoma and CPAs, **(D)** *CYP11B2* was highly expressed in the left adenoma and APAs compared with other adrenal diseases and nonfunctional adenoma. * *P* < 0.05 compared with other groups. NFA, nonfunctional adenomas; left ACA, left adrenocortical adenoma of the index patient; right ACA, right adrenocortical adenoma of the index patient; APA, aldosterone-producing adenomas; CPA, cortisol-producing adenomas.

The mRNA level of the steroidogenic enzyme STAR was high in both adenomas. CYP11B1 and CYP17A1 were highly expressed in the right adenoma and CPAs, whereas CYP11B2 was highly expressed in the left adenoma and APAs compared with other adrenal diseases and nonfunctional adenoma (**Figure 2**).

### Calcium channel involved in the secretion of aldosterone and cortisol

**Figure 3:**
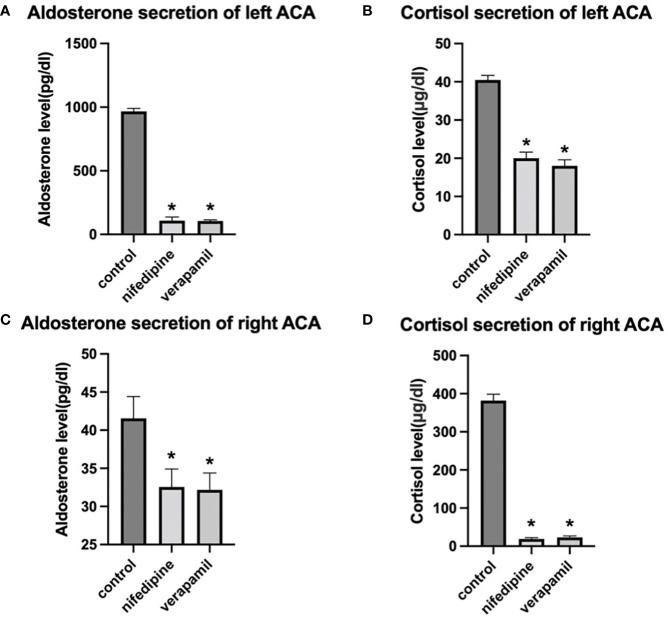
Effects of nifedipine and verapamil on aldosterone and cortisol secretions, respectively, in the primary cells of bilateral adenomas. **(A, B)** Effects of verapamil and nifedipine on the secretion of aldosterone and cortisol in the left adenoma. **(C, D)**. Effects of verapamil and nifedipine on the secretion of aldosterone and cortisol in right adenoma. * *P* < 0.05 compared with the control. left ACA, left adrenocortical adenoma of the index patient; right ACA, right adrenocortical adenoma of the index patient.

The bilateral adenoma cells were cultured *in vitro*, and the calcium channel blockers nifedipine and verapamil were administered as an intervention (classified into three groups: control, nifedipine, and verapamil groups). We further detected the concentrations of aldosterone and cortisol in the supernatant of the cultured cells. Aldosterone secreted by cultured cells of the left adenoma was significantly higher than that secreted by the cells of the right side. In contrast, cortisol secreted by the cultured cells of the right adenoma was significantly higher than that secreted by the cells of the left side. Moreover, verapamil and nifedipine remarkably inhibited the secretion of bilateral aldosterone and cortisol (**Figure 3**).

### Germline and somatic variant analysis

**Figure 4:**
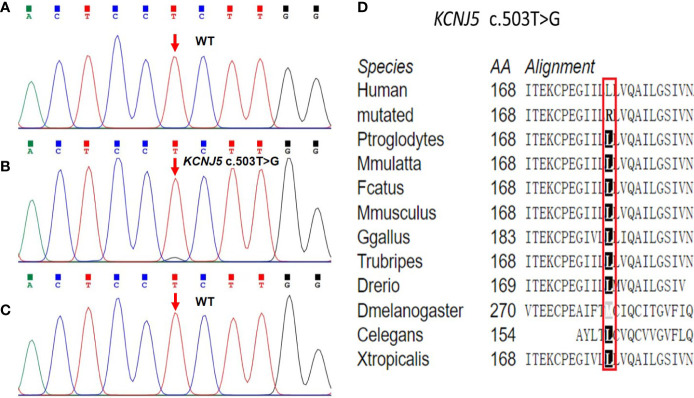
Sequencing chromatograms of the *KCNJ5* variant identified in peripheral blood samples and bilateral adenomas. **(A)** The germline *KCNJ5* variant was not found in peripheral blood samples. **(B)** Heterozygous *KCNJ5* c.503T > G somatic variant was identified in the left adenoma. **(C)** Normal sequence of the *KCNJ5* gene in the right adenoma. **(D)** Multiple alignment of the KCNJ5 protein sequence in different species, indicating conservation of the residues *KCNJ5* c.503T > G affected by this variant.

A novel somatic heterozygous missense variant, *KCNJ5* c.503T > G (p.L168R), was detected in the left adrenal adenoma, whereas no germline and somatic variants associated with PA and CS were found in the peripheral blood samples or right adrenocortical adenoma (**Figures 4A–C**). The amino acid alignment of KCNJ5 across different species revealed that the leucine residue mutated in our case was conserved across all examined species (**Figure 4D**). According to the ACMG guidelines, the KCNJ5 c.503T > G variant is considered pathogenic because: 1) this variant was found in an extremely low frequency (PM2), 2) it was predicted to be damaging and disease-causing by SIFT, PolyPhen-2, and MutationTaster (PP3), and 3) it was consistent with the patient’s phenotype (PP4).

## Discussion

PAs are rarely associated with subclinical or clinical CS. Spath et al. reviewed the clinical features of 34 patients with A/CPA and reported that the average age of participants was 52 years and more than two-thirds of the participants were females (19). The majority of patients with A/CPA presented with severe hypertension and electrolyte disturbances, 14% of A/CPA patients presented with typical symptoms of hypercortisolism, and 74% presented with preclinical CS. Among the 34 A/CPA cases, 29 had a single adenoma and only 5 had multiple lesions. In our study, the female patient had co-existing PA and clinical Cushing syndrome as well as bilateral adrenocortical adenomas of >2 cm. She developed diabetes mellitus and multiple target organ damage (including cardiomegaly, heart failure, proteinuria, and impaired renal function). This finding was consistent with that of previous reports on patients with A/CPA presenting with an increased risk for cardiovascular events, glucose intolerance, and postoperative adrenal insufficiency (19, 20). Given the adverse metabolic risk in A/CPA, the presence of an aldosterone and cortisol co-secreting adrenocortical tumor should be considered if a patient has PA and an adenoma of >2 cm or cortisol that is non-suppressible with overnight 1-mg DST.

The majority of the reported A/CPA tumors secreted aldosterone and cortisol simultaneously in the same tumor. Here we report an extremely rare case of bilateral adrenocortical adenomas secreting cortisol and aldosterone from each side. To date, only six cases of bilateral adenomas with different functions have been reported (**Table 3**) (21–26). Moreover, it is still challenging to detect the lateralization of A/CPA. Currently, the AVS lateralization index (aldosterone/cortisol of bilateral adrenal vein) has been widely used for the lateralizing functioning of primary aldosteronism. However, the secretion of cortisol is uneven in A/CPA tumors, which may lead to false-negative aldosterone-to-cortisol ratios. Thus, epinephrine levels in bilateral adrenal vein and peripheral vein were recommended to be used as the denominator for correcting aldosterone from the predominant side. Among the six reported cases of bilateral functioning adenomas (**Table 3**), only two cases underwent testing for epinephrine for correction in AVS. The remaining four cases did not undergo testing for epinephrine during AVS and were further confirmed by postoperative clinical manifestations, changes in hormone levels, immunohistochemical staining for steroidogenic enzymes, etc. The limitation of our study was the absence of epinephrine correction in interpretation the results of AVS. Nevertheless, bilateral adenomas with different function were confirmed by subsequent immunohistochemical analysis and *in vitro* experiments after the sequential removal of adrenal adenomas. Therefore, further studies with larger samples are required for more accurate localization.

**Table 3:** Clinical features of case reports published with bilateral adrenocortical adenomas secreting cortisol or aldosterone independently.

Study ID	Age	Gender	Hypokalemia	ACTH (pg/ml)	COT (μg/dl)	ALD (pg/ml)	PRA (ng/mL/h)	CS	Left lesion (mm)	Right lesion (mm)	Operation choice
Nagae A. (21)	55	F	Y	14.1	17.3	242	1.4	Y	two CPAs 8, 20	APA 9	Right partial adrenalectomy, left total adrenalectomy
Oki K. (22)	50	M	Y	21.6	16.5	203	1.1	N	CPA 10	APA 10	Left partial adrenalectomy, right total adrenalectomy
Onoda N. (23)	43	F	Y	ND	29.4	305	1.5	Y	APA 20	CPA 30	Bilateral partial adrenalectomy
Morimoto R. (24)	54	F	N	ND	24	128	0.1	Y	APAs, 2 micro nodules	CPA 30	Right partial adrenalectomy, left total adrenalectomy
Seung-Eun Lee. (25)	31	F	Y	20.1	6.8	244.0	0.76	N	CPA 20	APA 16	Right partial adrenalectomy, left total adrenalectomy
Ren K. (26)	30	M	Y	17.8	12.5	259	2.0	N	CPA 25	APA 19	Bilateral partial adrenalectomy

All six patients were diagnosed based on adrenal venous sampling (AVS).

COR, plasma cortisol level; UFC, urinary free cortisol; ALD, plasma aldosterone level; PRA, plasma renin activity; CPA, corticoid producing adenoma; APA, aldosterone producing adenoma; CS, Cushing’s syndrome; ND, undetectable. F, female; M, male; Y, yes.

The exact mechanisms of tumorigenesis in A/CPA remain to be fully elucidated. As reported previously, APAs composed of pure adrenal cortical zonal cells are very rare, and most APAs are composed of different cell types. Thus, APA tumors have the potential to co-secrete aldosterone and cortisol. Previous studies have reported the capacity of APA cells to produce cortisol (27, 28). Spath et al. reported two different morphological cell types in A/CPA tumors: zona glomerulosa-like cells and zona fasciculate-like cells. Immunohistochemical analysis indicated that A/CPA tumors could express CYP11B1 and CYP11B2—the key enzymes for synthesizing aldosterone and cortisol—simultaneously, but the composition ratio of these two cells and the expression of the two synthases were different in addition to the presence of marked heterogeneity in the tumors (19). In our case, immunohistochemical analysis and mRNA levels of steroidogenic enzymes revealed a left adenoma that primarily consisted of clear cells and mainly expressed CYP11B2, whereas the right adenoma was composed of both eosinophilic compact and clear cells and mainly expressed CYP11B1. The different cell composition of the bilateral adrenocortical adenomas may be an important reason for the secretion of different hormones in each adrenal gland.

A recent Chinese study reported that the *KCNJ5* mutation was detected in 9 of 11 A/CPA cases, and *PRKACA* gene mutation was detected in two A/CPA cases. Another study detected 17 samples harboring *KCNJ5* mutations among the 22 patients with A/CPA, but *PRKACA*, *CACNA1D*, *CACNA1H*, *ATP1A1*, or *ATP2B3* mutations were not detected (5). Thus, the authors of that study suggested that A/CPA was more similar to APA than CPA (5, 29). In our case, we identified a novel somatic *KCNJ5* c.503T > G mutation in the left adrenocortical adenoma. However, no germline and somatic variants associated with PA and CS were found in the peripheral blood samples or right adrenal adenoma. This observation may also explain significantly higher aldosterone levels in the left adrenal adenoma than those in the right adrenal adenoma.

Elevated cytoplasmic Ca^2+^ concentrations have been reported to be critical in the pathogenesis of APA with a mutant *KCNJ5*, and Ca^2+^ channel blockers can reduce aldosterone secretion in H295R cells expressing mutant *KCNJ5* (30, 31). Meanwhile, blockers of voltage-gated Ca^2+^ channels can inhibit ACTH-stimulated Ca^2+^ influx and consequent cortisol secretion (32). In the present study, bilateral adenoma cells were cultured *in vitro*, and the L-type Ca^2+^ channels blockers nifedipine and verapamil were administered for intervention. We found that both verapamil and nifedipine reduced the secretion of bilateral aldosterone and cortisol, suggesting that hypersecretion of aldosterone and cortisol in our patient was mediated by voltage-gated Ca^2+^ channels.

## Conclusions

We report an extremely rare case of bilateral adrenocortical adenomas with distinct secretion of aldosterone and cortisol, as confirmed by clinical findings and pathological studies. The heterogeneity of the tumor cell compositions of A/CPAs and somatic mutation of *KCNJ5* may have led to different hormone secretions in the bilateral adrenal adenomas.

## Data availability statement

The data presented in the study are deposited in the BioProject repository, and the BioProject ID is PRJNA923486.

## Ethics statement

The studies involving human participants were reviewed and approved by the institutional review board of Third Xiangya Hospital, Central South University, China. The patients/participants provided their written informed consent to participate in this study. Written informed consent was obtained from the individual(s) for the publication of any potentially identifiable images or data included in this article.

## Author contributions

Study concepts were prepared by PJ. The study was designed by PJ and LZ. Data acquisition was performed by LZ, JJW, and YW. Statistical analysis was done by LZ and WY. Data analysis and interpretation was done by LZ and PJ. AVS was performed by QL. Bilateral partial adrenal resection was performed by JRW. The manuscript was prepared and edited by LZ and PJ. All authors contributed to the article and approved the submitted version.
