# Supplementary material for: Different cell compositions and a novel somatic KCNJ5 variant found in a patient with bilateral adrenocortical adenomas secreting aldosterone and cortisol
Source: Front Endocrinol (Lausanne). 2023 Mar 7;14:1068335. doi: 10.3389/fendo.2023.1068335 (PMC10028271; doi:10.3389/fendo.2023.1068335)
Supplement: Supplementary file 1 [file Table_1.docx]

Supplemental Table 1. Results of AVS

|  | Left adrenal vein | | | | IVC | | | | Right adrenal vein | | | |  | |
| --- | --- | --- | --- | --- | --- | --- | --- | --- | --- | --- | --- | --- | --- | --- |
|  | Aldost.  (pg/mL) | Cortisol  (μg/dL) | A/C | Aldost.  (pg/mL) | | Cortisol  (μg/dL) | A/C | Aldost.  (pg/mL) | | Cortisol  (μg/dL) | A/C | LI | | Cortisol |
|  |  |  |  |  |  |  |  |  |  |  |  | L/R | | R/L ratio |
| −10’ | 93590.0 | 285.0 | 328.4 | 1094.6 | | 24.8 | 40.8 | 1600.7 | | 1032.4 | 1.6 | 205.3 | | 3.6 |
| −5' | 94576.0 | 295.0 | 320.6 | 1149.6 | | 36.6 | 31.4 | 1426.3 | | 932.8 | 1.5 | 213.7 | | 3.2 |
| 10’ post ACTH | 132587.0 | 427.0 | 310.5 | 1202.9 | | 36.9 | 32.6 | 5716 | | 4141.7 | 1.4 | 221.8 | | 9.7 |
| 20’ post ACTH | 152230.0 | 1390.0 | 109.5 | 1336.4 | | 30.6 | 30.6 | 5863 | | 4297.2 | 1.4 | 78.2 | | 3.1 |

AVS, adrenal vein sampling; Aldost., aldosterone; IVC, inferior vena cava; A/C, aldosterone/cortisol; ACTH, adrenocorticotropic hormone; LI, lateralization index.
